# Seasonality of influenza and its association with meteorological parameters in two cities of Pakistan: A time series analysis

**DOI:** 10.1371/journal.pone.0219376

**Published:** 2019-07-19

**Authors:** Nadia Nisar, Nazish Badar, Uzma Bashir Aamir, Aashifa Yaqoob, Jaya Prasad Tripathy, Chinmay Laxmeshwar, Fariha Munir, Syed Sohail Zahoor Zaidi

**Affiliations:** 1 National Influenza Center, Department of Virology, Public Health Laboratories Division, National Institute of Health, Islamabad, Pakistan; 2 National TB Control Program (NTP), Ministry of National Health Services Regulation & Coordination, Government of Pakistan, Islamabad, Pakistan; 3 International Union against Tuberculosis and Lung Diseases, The Union South East Asia Office, New Delhi, India; 4 International Union Against Tuberculosis and Lung Disease, Paris, France; 5 Medecins SansFrontieres, Mumbai, India; Stanford University School of Medicine, UNITED STATES

## Abstract

**Background:**

Influenza is known to have a specific pattern of seasonality the reasons for which are yet to be fully ascertained. Temperate zones show influenza epidemic during the winter months. The tropical and subtropical regions show more diverse influenza outbreak patterns. This study explores the seasonality of influenza activity and predicts influenza peak based on historical surveillance time series data in Islamabad and Multan, Pakistan.

**Methods:**

This is a descriptive study of routinely collected monthly influenza sentinel surveillance data and meteorological data from 2012–16 in two sentinel sites of Pakistan: Islamabad (North) and Multan (Central).

**Results:**

Mean number of cases of influenza and levels of precipitation were higher in Islamabad compared to Multan. Mean temperature and humidity levels were similar in both the cities. The number of influenza cases rose with decrease in precipitation and temperature in Islamabad during 2012–16, although the same cannot be said about humidity. The relationship between meteorological parameters and influenza incidence was not pronounced in case of Multan. The forecasted values in both the cities showed a significant peak during the month of January.

**Conclusion:**

The influenza surveillance system gave a better understanding of the disease trend and could accurately forecast influenza activity in Pakistan.

## Introduction

Influenza is a communicable respiratory disease caused by the influenza virus. The disease severity may range from mild to severe, sometimes leading to fatality, especially among infants, elderly, immune-compromised persons and various other high risk groups.The World Health Organization (WHO) has reported 3–5 million cases of severe illness and 290,000–650,000 deaths worldwide in 2017 due to seasonal influenza epidemics [1].

Influenza is known to have a specific pattern of seasonality the reasons for which are yet to be fully ascertained [2]. Studies report that temperate zones show influenza epidemic during the winter months of November-March in northern hemisphere and May-September in southern hemisphere [3,4]. The tropical and subtropical regions show more diverse influenza outbreak patterns. Annual epidemics occur in the tropical locations that usually coincide with the rainy season, while biannual incidence is the norm in some regions, and influenza activity occurs throughout the year in other countries [3]. There is limited understanding about the epidemiology of influenza and the characteristics of seasonal influenza in the tropical region where there is influenza activity all throughout the year.[5,6]

A number of theories have been proposed over the years to explain the seasonality of these epidemics [7]. These consider that cool and dry surroundings are the reason for this [8]. In tropical and subtropical regions the upsurge of influenza has been coupled with precipitation (rain). Epidemics also occur in these regions in other seasons, when the atmospheric conditions are humid and hot [4]. In some sub-tropical and tropical regions no significant relation between rainfall and influenza activity has been observed [9].

There are three mediating mechanisms that might help in explaining influenza activity and epidemics: 1) Contact rate, 2) Viral survival and 3) Host immunity [4]. Climatic conditions like humidity, temperature and precipitation play a great role in elevating viral survival and activity. It has been speculated that low indoor humidity, cool temperature and less solar radiations support viral activity, especially during the winter epidemics in the temperate zone. Recent evidence shows that rainy season favours viral survival and disease transmission in tropics [10,11]. It is possible that the seasonal factors that govern activity of influenza virus in temperate zones do not impact influenza virus very strongly in the tropics. [3]

Pakistan is situated in South-East Asia with tropical to temperate climatic conditions and arid environment in the southern region. The country had a robust laboratory-based influenza sentinel surveillance system in eight sites from 2009–2016. This provided an opportunity to study the relationship between meteorological parameters (humidity, precipitation and temperature) and influenza activity in two major cities of Pakistan, Islamabad and Multan in the post 2011 pandemic period. We also sought to explore the seasonality of influenza activity and predict influenza peak based on historical surveillance time series data.

## Materials and methods

### Study design

This is a descriptive study of routinely collected monthly influenza sentinel surveillance and meteorological data from 2012–16 in two geographical sentinel sites of Pakistan: Islamabad (North) with geo-coordinates of latitude: 33.738 and longitude: 73.084and Multan (Central) with geo-coordinates of latitude: 30.181and longitude: 71.492.

### Setting

#### General setting

Pakistan is the fifth most populous South Asian country [12]. It has a population of over 200 million according to the United Nations estimates [13]. The country has a tropical to temperate climate and arid conditions in the coastal south. Rainfall varies greatly from year to year, and patterns of alternate flooding and drought are common.[14]

The climate of Islamabad is humid subtropical type with five distinct seasons: winter (November−February), spring (March−April), summer (May−June), monsoon (July−August) and autumn (September−October). June is the hottest with temperature routinely soaring above 38°C (100.4°F). July witnesses heavy rainfall whereas January is cold with temperatures usually dropping below zero degrees[15].

Multan is located in the southern part of Punjab province in Pakistan. Multan features an arid climate with very hot summers and cold winters. Multan has four seasons: winter (December-February), summer (May-Sep), autumn (Oct-Nov) and spring (Mar-Apr) without a rainy season[14]. The city witnesses some of the most extreme temperatures in the country. Being close to the deserts, it has seen some of the worst heat waves in the history of Pakistan with scanty rainfall.

#### Influenza surveillance system

In 2008, a sentinel laboratory-based influenza surveillance network was established in collaboration with the US-Centers for Disease Control and Prevention (CDC). The Federal Government Services Hospital in Islamabad was the first sentinel site under the network [16]. Seven more influenza surveillance sentinel sites in high load tertiary hospitals across four provinces and two regions have been set up since then. This includes the Nishter Medical Collage and Hospital Multan in 2012.

In each of the study sites, presumptive cases i.e. Influenza like Illness (ILI) or Severe Acute Respiratory Illness (SARI) were referred by the physicians (from the departments of Medicine, Pediatrics, Ear Nose and Throat and Obstetrics and Gynecology) to the project laboratory technician for throat/nasopharyngeal swab collection. The laboratory staff and other project staff were trained in sample processing and testing, data entry and database maintenance. The sample collected was then sent to the virology laboratory for testing. The demographic and clinical data were recorded at the time of sample collection and also validated by cross-checking with the hospital patient records.

Laboratory testingThroat and/or nasopharyngeal swabs collected from suspected cases in 2–3 ml viral transport medium (Virocult) were stored at -70°C before use. RNA was extracted from samples using QiagenQIAmp Viral RNA mini kit and eluted in 60ul elution buffer. The samples were analyzed by one-step real-time reverse transcription-polymerase chain reaction (rRT-PCR) on Applied Biosystems platform ABI 7500 following recommended US-CDC protocol. The assay was performed using AgPath-IDTM One–Step RT-PCR (Ambion; California, USA). Briefly, 25ul PCR mixture containing 0.5ul each of probe (FAM labeled), forward and reverse primers, 1ul enzyme mix, 12.5 ul of 2X master mix, 5 ul nuclease-free water and 5ul of extracted RNA was subjected for amplification with conditions; reverse transcription at 50°C for 30 min, Taq inhibitor activation 95°C for 10 min and 45 cycles at 95°C for 15s, extension step of 55°C for 31s[16].

#### Case definitions

ILI case is defined as a person with acute respiratory illness, with temperature of (>38°C), and cough within past ten days of onset. SARI is defined as those with acute respiratory illness, measured fever of (>38°C), cough within past ten days of onset and requiring hospital admission[17].

#### Study variables

Data on influenza cases and positivity were obtained from the surveillance database during 2012–16. Surveillance data during the pandemic influenza seasons (2009–2011) were excluded to understand the characteristics of seasonal influenza only. Monthly data on three weather parameters namely temperature, precipitation and humidity were obtained from the Pakistan MeteorologicalDepartmentat(: *http*:*//www*.*pmd*.*gov*.*pk/*).

#### Data analysis

Data were entered into IBM SPSS (version 22.0, Chicago, USA) version and analyzed. Line graphs were used to present the monthly trend in the number of confirmed influenza cases and its relationship with other meteorological parameters for both the sentinel sites separately during 2012–16. Correlation coefficient was reported to measure the strength and direction of association between meteorological parameters and influenza cases and test positivity at monthly intervals.

#### Fitting a time series model

We used exponential smoothing models to describe autocorrelations in the data and forecast influenza activity for the next 12 months [18]. Expert modeler of SPSS version 21 was used to choose the best fitting model for the time series data. The Expert Modeler tries to identify the best-fitting ARIMA or exponential smoothing model, thus eliminating the need to identify an appropriate model through trial and error.Seasonal adjusted factor (SAF) was used to measure seasonal variation and identify the seasonal peak. The Ljung-Box Q test statistic was used to determine if the model fits well with the data.The autocorrelation function (ACF) plots of the residuals and Ljung-Box tests were used to determine if there is any autocorrelation in the residuals [19].

#### Ethical considerations

The Union Ethics Advisory Group, Paris, France granted ethical approval for the study. Administrative approval was obtained from the National Institute of Health, Islamabad, Pakistan. As the study involved review of existing project database, a waiver of informed consent was granted.

## Results

Table 1 provides a description of the meteorological parameters (precipitation, humidity and temperature) and the influenza activity in Islamabad and Multan. The mean number of cases of influenza and levels of precipitation were much higher in Islamabad compared to Multan. Mean temperature and humidity levels were similar in both the cities.

**Table 1:** Meteorological parameters (humidity, precipitation and temperature) and number of influenza cases in two cities of Pakistan, 2012–16.

Characteristics	Mean	Standard deviation	Min	Max	P25	P50/ Median	P75
**Islamabad**							
Influenza cases	16	37	0	261	.25	3	14
Humidity (%)	48	21	6	86	32	50	63
Precipitation (in mm)	101	124	0	483	21	45	143
Temperature (^o^C)	24	8	11	41	17	25	30
**Multan**							
Influenza cases	3	4	0	15	0.0	1	2
Humidity (%)	48	17	4	71	31	53	59
Precipitation (in mm)	28	31	0	98	0.3	16	31
Temperature (^o^C)	24	8	13	36	19	24	50

**Fig 1:**
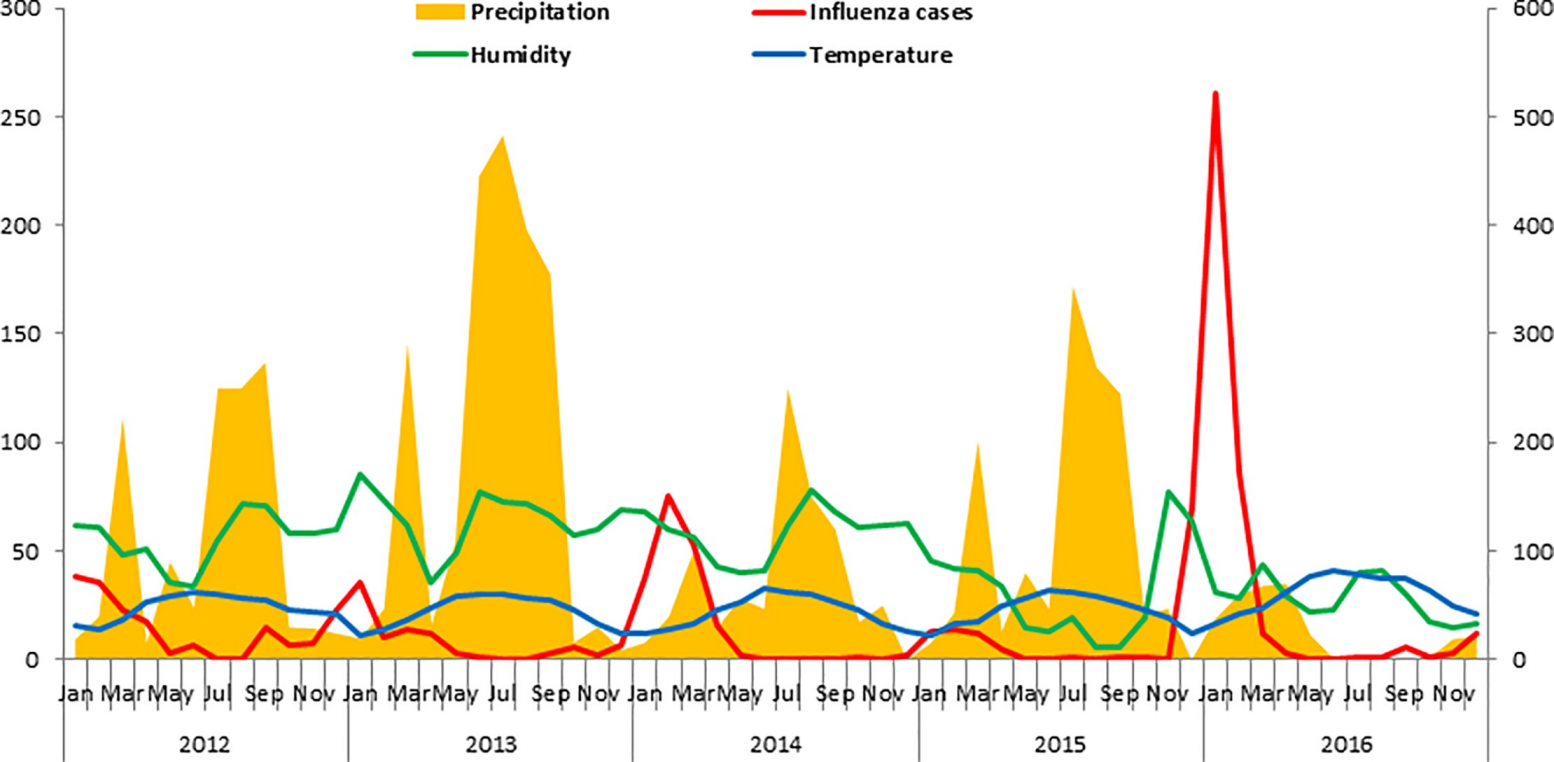
Precipitation, humidity, temperature and influenza cases in Islamabad during 2012–16.

**Fig 2:**
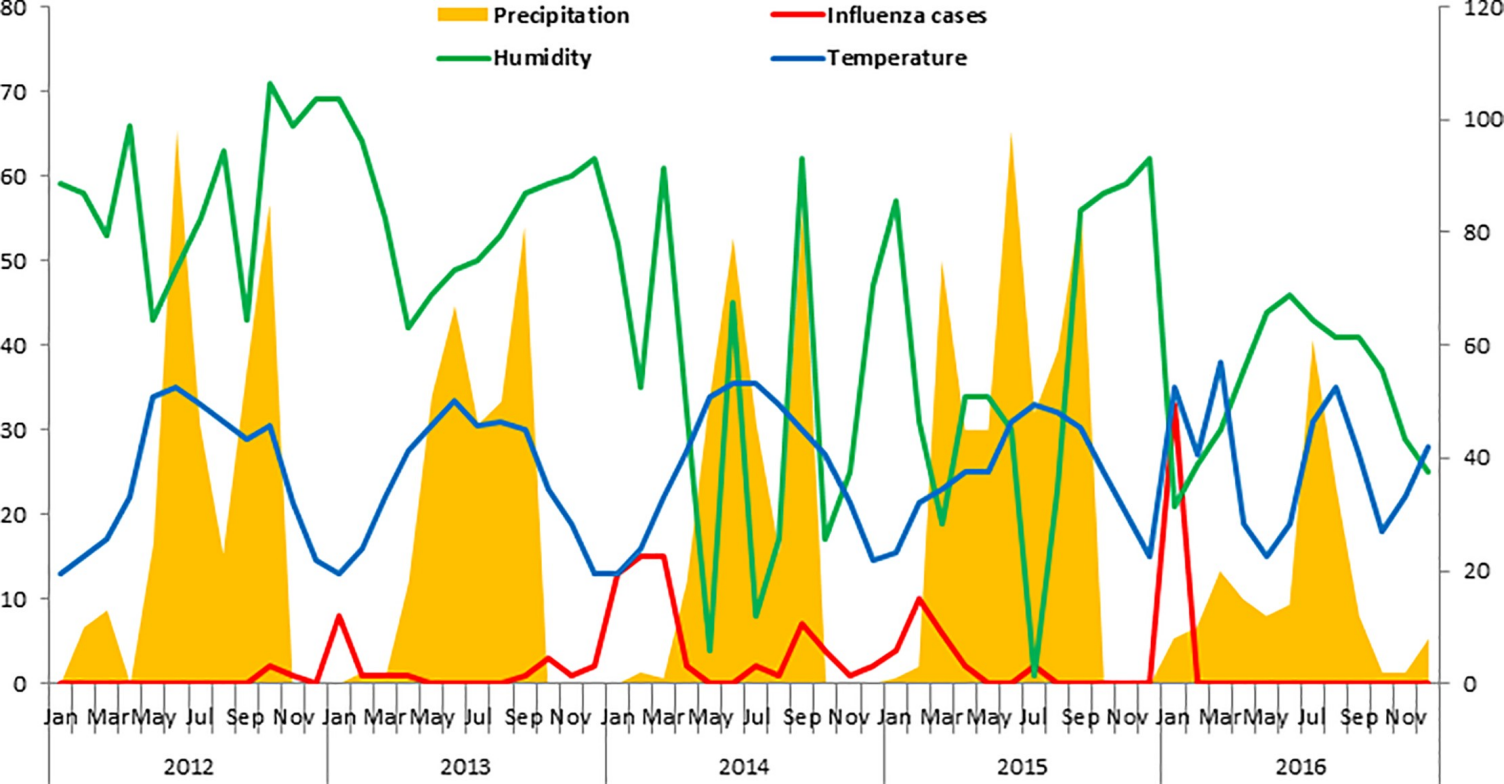
Precipitation, humidity, temperature and influenza cases in Multan during 2012–16.

Fig 1 shows that the number of influenza cases rose with decrease in precipitation and temperature in Islamabad during 2012–16, although the same cannot be said about humidity. The relationship between meteorological parameters and influenza incidence was not pronounced in case of Multan (Fig 2).

Overall, temperature (r = -0.42, *p* = 0.001) and precipitation (r = -0.3, *p* = 0.02) was found to be significantly correlated with test positivity whereas temperature (-0.3, *p* = 0.002) alone was correlated with the number of cases of influenza, similar findings were also seen in Islamabad. However, no significant correlation was found between meteorological parameters and influenza cases in Multan. (Table 2).

**Table 2:** Correlation of meteorological parameters with influenza test positivity and number of cases of influenza in two cities of Pakistan, 2012–16.

Overall (n = 120)	Test positivity	*p*-value	Number of cases	*p*-value
Temperature	-0.42	0.001**	-0.3	0.002**
Humidity (%)	0.02	0.88	0.01	0.9
Precipitation (mm)	-0.3	0.02*	-0.057	0.54
**Islamabad (n = 60)**				
Temperature	-0.42	0.001**	-0.38	0.003**
Humidity (%)	0.02	0.8	-0.01	0.8
Precipitation (mm)	-0.3	0.02*	-0.17	0.19
**Multan (n = 60)**				
Temperature	-0.15	0.36	-0.07	0.6
Humidity (%)	-0.1	0.53	-0.11	0.4
Precipitation (mm)	-0.16	0.34	-0.19	0.15

**correlation significant at 0.01 level

* correlation significant at 0.05 level

**Fig 3:**
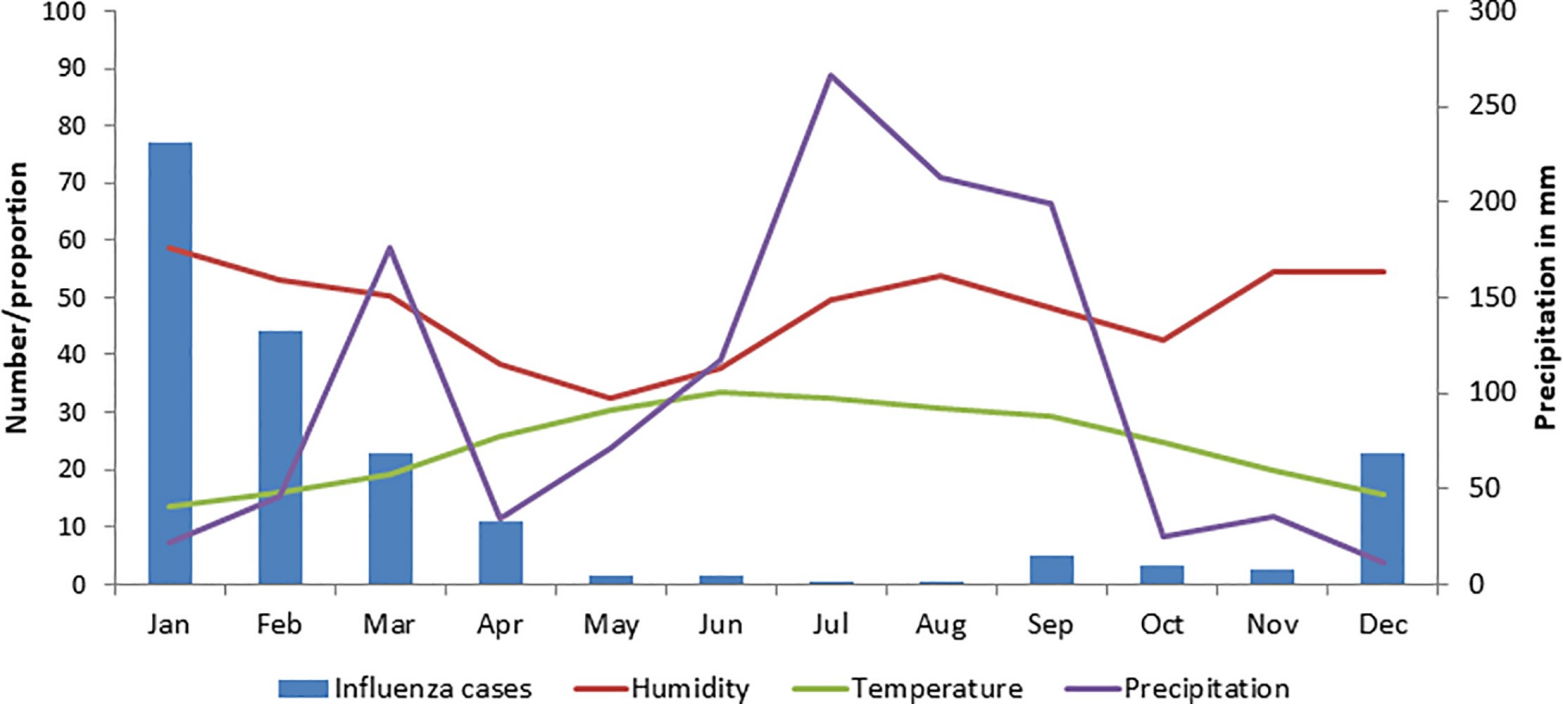
Association of precipitation, humidity and temperature with number of influenza cases in Islamabad and Multan during 2012–16.

Fig 3 also shows the relationship between meteorological parameters and influenza cases in each month. An increase in cases of influenza is seen with a fall in temperature and precipitation.

Exponential smoothing models were applied. Winter’s additive model was the best fitted mathematical model for the time series data of Multan whereas simple seasonal model fitted well with the influenza surveillance data for Islamabad. The Ljung-Box (modified Box-Pierce) test indicated that the model was correctly specified. The expert modeler detected no outliers in the data.(Table 3).

**Table 3:** Model statistics for influenza surveillance data in two cities of Pakistan, 2012–16.

	Model fit statistics	Ljung Box Q			Model type
	Stationary R-squared	Statistics	df	*p*-value	
**Islamabad**	0.63	18.3	16	0.607	Simple seasonal
**Multan**	0.70	6.9	15	0.960	Winter’s additive

**Fig 4:**
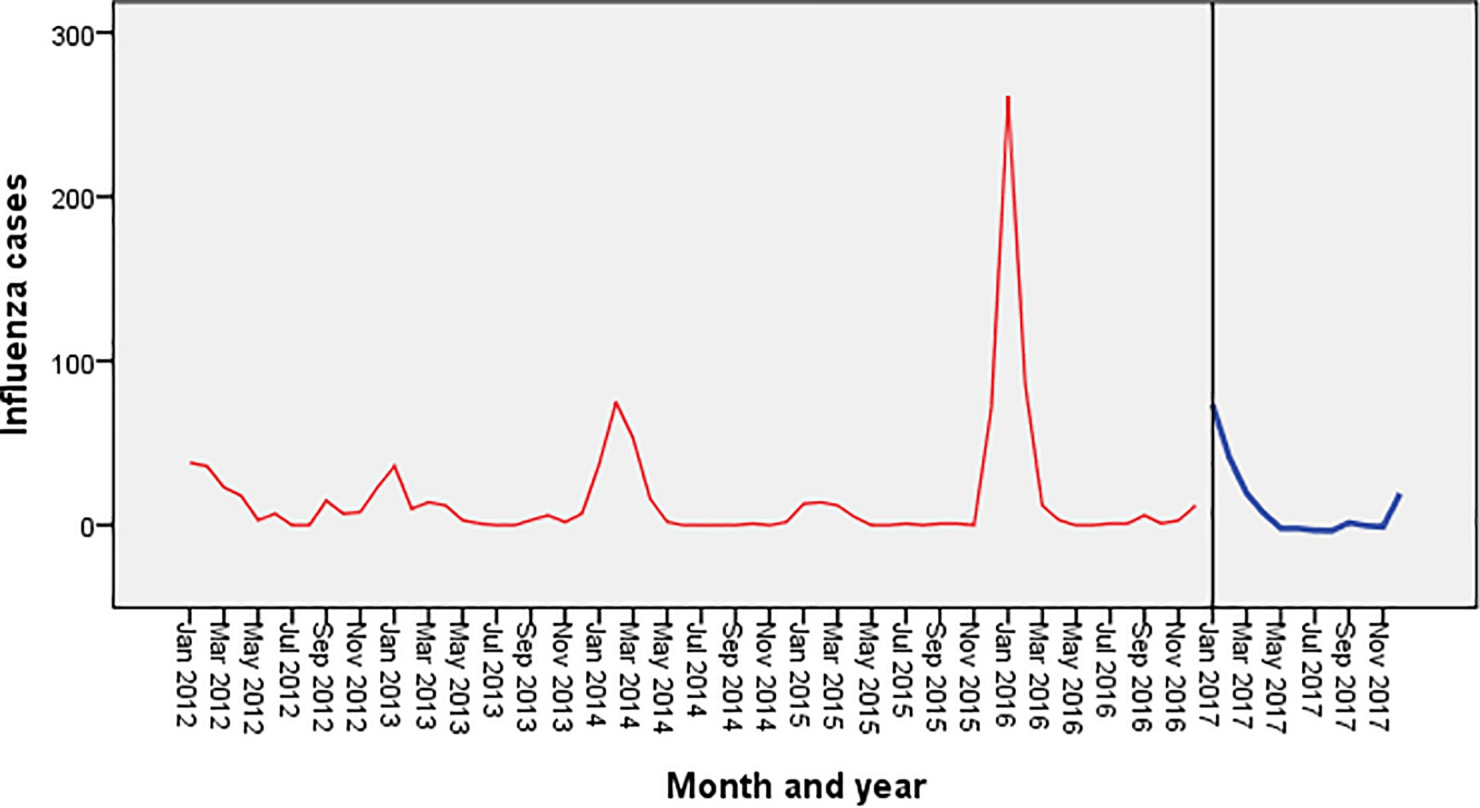
Forecasting of influenza in Islamabad using exponential smoothing model. The blue line represents the observed values of influenza incidence from 2012 to 2016, and the red line represents the constructed model’s fitted curve of 2017.

Fig 4 shows the trend of influenza cases during the period 2012–16 in Islamabad. It shows seasonal peaks during the winter months (December-January) along with small peaks during the months of July-September.

**Fig 5:**
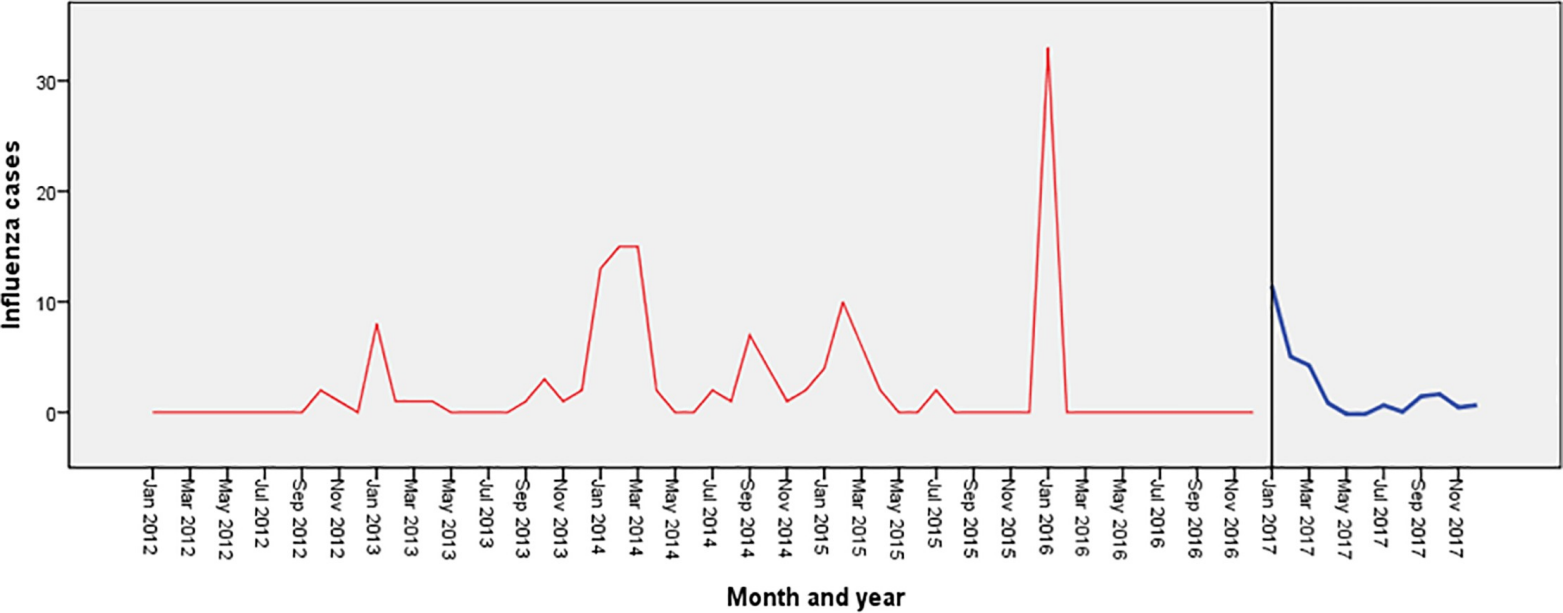
Forecasting of influenza in Multan using exponential smoothing model. The blue line represents the observed values of influenza incidence from 2012 to 2016, and the red line represents the constructed model’s fitted curve of 2017.

Fig 5 shows the seasonal trend of influenza cases during 2012–16 in Multan showing regular seasonal peaks during the winter months (December-February). No declining trend was seen in both the sequence charts.

The forecasted values in both the cities are shown in Figs 4 & 5 which depicts a significant peak during the month of January with no overall declining trends.

Of 6298 cases tested for influenza, 4783 (76%) were from Islamabad and remaining 1515 (24%) came from Multan. Overall, more ILI cases (3315, 53%) were tested compared to SARI (2983, 47%). The socio-demographic and clinical details of patients tested for influenza are given in Table 4.

**Table 4:** Socio-demographic and clinical characteristics of patients tested for influenza in two cities of Pakistan, 2012–16.

Characteristics	Islamabad	Multan	Total
	(n = 4783)	(n = 1515)	(n = 6298)
**Age groups**			
<2 years	593 (12.4)	538 (35.5)	1131 (18.0)
2–4 years	372 (7.8)	170 (11.2)	542 (8.6)
5–14 years	716 (15.0)	125 (8.3)	841 (13.4)
15–49 years	2150 (45.0)	551 (36.4)	2701 (42.9)
50–64 years	554 (11.6)	93 (6.1)	647 (10.3)
65 and above	398 (8.3)	38 (2.5)	436 (6.9)
**Gender**			
Male	2556 (53.4)	939 (62.0)	3495 (55.5)
Female	2227 (46.6)	576 (38.0)	2803 (44.5)
**Type of case**			
ILI^a^	2763 (57.8)	552 (36.4)	3315 (52.6)
SARI^b^	2020 (42.2)	963 (63.6)	2983 (47.4)
**Year**			
2012–2013	709 (14.8)	33 (2.2)	742 (11.8)
2013–2014	1033 (21.6)	393 (25.9)	1426 (22.6)
2014–2015	966 (20.2)	467 (30.8)	1433 (22.8)
2015–2016	636 (13.3)	488 (32.2)	1124 (17.8)
2016–2017	1439 (30.1)	134 (8.8)	1573 (25.0)

^a^ILI: Influenza like Illness

^b^SARI: Severe Acute Respiratory Illness

Of those tested, 1101 cases of influenza were reported from Islamabad (n = 961) and Multan (n = 140). Among confirmed cases of influenza, majority belonged to the age group 15–49 years (471, 49%) followed by 50–64 years (129, 13%) in Islamabad whereas in Multan most of the cases reported from age group 15–49 years (46,33%) followed by children<2 years (38, 27%). Most of the positive cases came from SARI from both the sites (n = 611, 55%). Influenza A/H1N1pdm09 was the predominant strain (41.9%, n = 403) followed by influenza A/H3N2 (273, 28%) in Islamabad. Multan also followed the same pattern and reported highest number of A/H1N1pdm09 (60, 44%) followed by influenza B (28, 20%) (Table 5).

**Table 5:** Socio-demographic and clinical characteristics of confirmed influenza cases in two cities of Pakistan, 2012–16.

Characteristics	Islamabad	Multan	Total
	n = 961	n = 140	n = 1101
**Age groups**			
<2 years	85 (9)	38 (27)	123 (11)
2–4 years	55 (6)	37 (26)	92 (8)
5–14 years	125 (13)	8 (6)	133 (12)
15–49 years	471 (49)	46 (33)	517 (47)
50–64 years	129 (13)	9 (6)	138 (13)
65 and above	96 (10)	2 (1)	98 (9)
**Gender**			
Male	543 (57)	97 (69)	640 (58)
Female	418 (43)	43 (31)	461 (42)
** Type of case**			
ILI^a^	452 (47)	38 (27)	490 (44)
SARI^b^	509 (53)	102 (73)	611 (56)
**Year**			
2012–2013	94 (10)	3 (2)	112 (10)
2013–2014	178 (19)	18 (13)	181 (16)
2014–2015	186 (19)	62 (44)	248 (23)
2015–2016	117 (19)	24 (17)	141 (13)
2016–2017	386 (40)	33 (24)	419 (38)
**Influenza Strains**			
A (not typed)	38 (4)	32 (23)	70 (6)
A/H1N1pdm09	403 (41.9)	60 (44)	463 (42)
A/H1N1	1 (0.1)	2 (1)	3 (1)
A/H3N2	273 (28)	18 (12)	291 (26)
B	246 (26)	28 (20)	274 (25)

^a^ILI: Influenza like Illness

^b^SARI: Severe Acute Respiratory Illness

**Fig 6:**
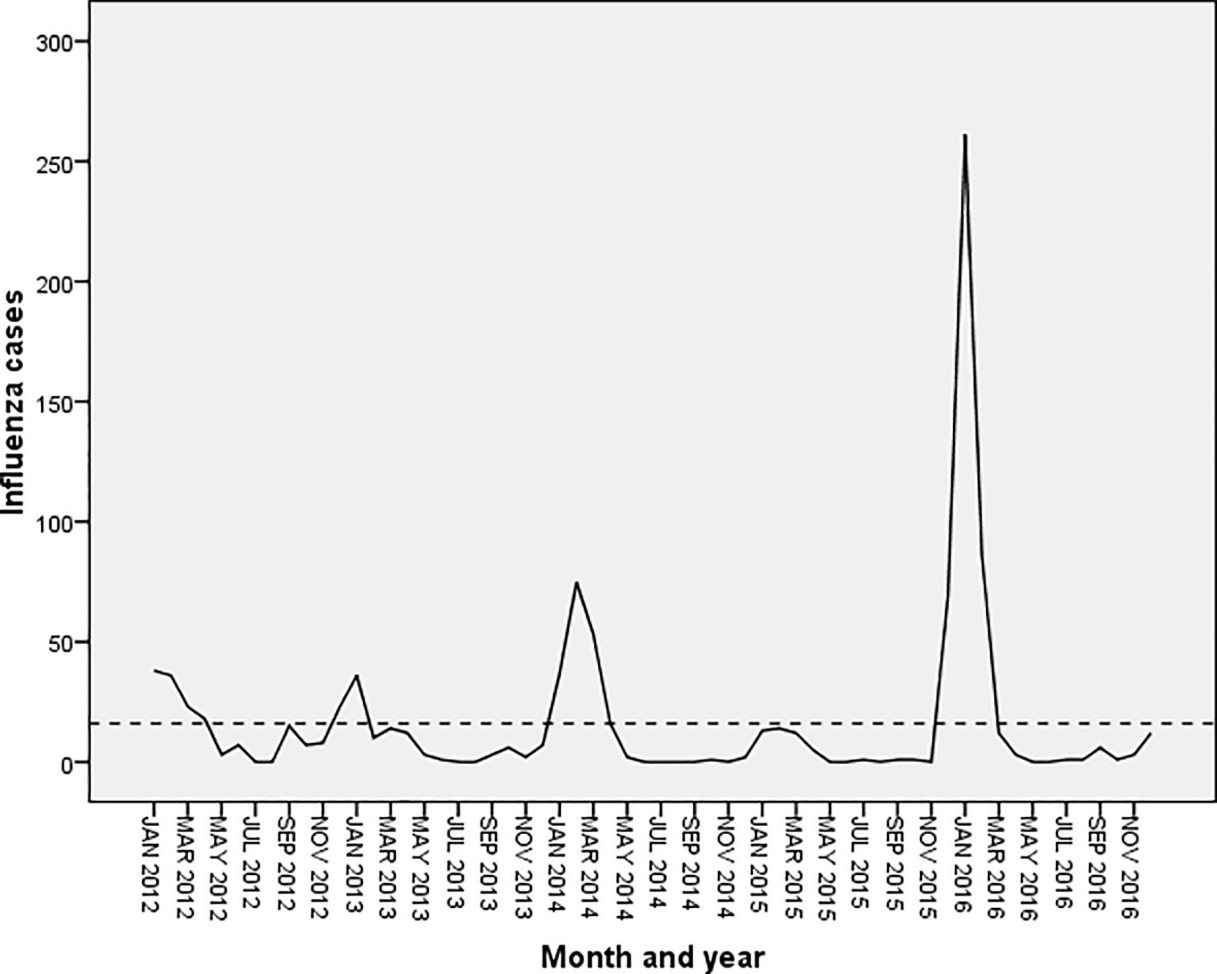
Weekly incidence curve of influenza in two cities of Pakistan, 2012–16.

Fig 6 shows that the cases of influenza peaked during the months of December, January and February with small peaks in the months of July and October in 2012.

## Discussion

This study highlights the relationship between meteorological parameters and influenza activity in two major cities of Pakistan in the post-2011 pandemic period and also predicts influenza activity based on historical surveillance time series data. Time series analysis of infections is useful to propose new hypotheses, predict epidemics and peaks, and improve the disease response system. This study used the exponential smoothing model to forecast influenza incidence, contributing to an early warning system that can help public health planners take appropriate preventive measures, improve public awareness, and tackle the situation effectively.

The association between influenza activity and climatic factors was assessed for 78 geographical locations around the globe. At high latitudes, influenza peaks coincided with months of lower temperature and lower humidity.In contrast, peak influenza activity in localities within 10° of the equator correlated with months of high specific humidity and precipitation.At intermediate latitudes such as Pakistan, no significant association was observed which somewhat supports the findings of this study[20–23].

The study had some interesting findings. First, influenza activity, in terms of number of cases and test positivity both negatively correlated with temperature. This finding is consistent with studies from Portland and New York in the United States. [24,25]. On the other hand, studies in Thailand and the tropical Central American countries like Guatemala, El Salvador and Panama with an average temperature of 25–29 ^o^C showed a positive correlation with temperature [26].

Second, in contrast to the studies elsewhere, no significant correlation was found between humidity levels and influenza activity [26]. However, amulti-site influenza study from India reported a positive correlation between relative humidity and influenza positive cases by multivariate analysis[27]. Another study from Thailand showed that only maximum relative humidity correlated with suspected influenza cases in the southern region, whereas all parameters including maximum, minimum, and average relative humidity correlated with suspected influenza cases in the central region[28].

Third, the study forecasted significant peak during the month of January with no declining trend [26,29]. This could be useful information for the administrators to prepare for the peak and mitigate it effectively.

The study has some significant strong points. First, the influenza surveillance data were part of a US-CDC supported laboratory based sentinel surveillance project in tertiary care hospitals across the country with high patient influx. Confirmation of diagnosis of influenza was done using real time RT-PCR which is the gold standard test. Second, five-year historical data (2008–17) on influenza surveillance in two major cities of the country allows a better understanding of the trend and accurate forecasting of the disease. The quality and consistency of the surveillance data is likely to produce meaningful predictions for policy decisions.

The study had some limitations as well. First, the cases of influenza presented in this study were reported from hospital-based sentinel surveillance sites, thus, missing out an unknown proportion of cases in the community who could not visit the facilities due to poor access or did not require hospital care due to mild symptoms or visited the private sector and other levels of health care. Second, the prediction models are generally used for short-term forecasts because the relative bias of prediction increases with time. This might be explained by antigenic drift of the influenza virus and many unknown factors that affect disease transmission. Third, we could only infer associations, but not causality, between influenza activity and various meteorological parameters. As a result, the associations we found may act only as proxies for factors not considered in this study.

## Conclusion

In conclusion, the influenza surveillance system gave a better understanding of the disease trend and could accurately forecast influenza activity in Pakistan. Exponential smoothing model was the best fit statistical model for predicting influenza cases. This information will be useful for public health administrators in effectively implementing preventive and control measures for the seasonal influenza outbreaks or epidemics.

## Supporting information

S1 File Climate SPSS for means. (SAV)

S2 File Climate data-average. (XLSX)

S1 Fig Suppl Fig 1. (PPTX)
