# Supplementary figures and images for: Seasonality of influenza and its association with meteorological parameters in two cities of Pakistan: A time series analysis
Source: PLoS One. 2019 Jul 19;14(7):e0219376. doi: 10.1371/journal.pone.0219376 (PMC6641468; doi:10.1371/journal.pone.0219376)

## Slide 1
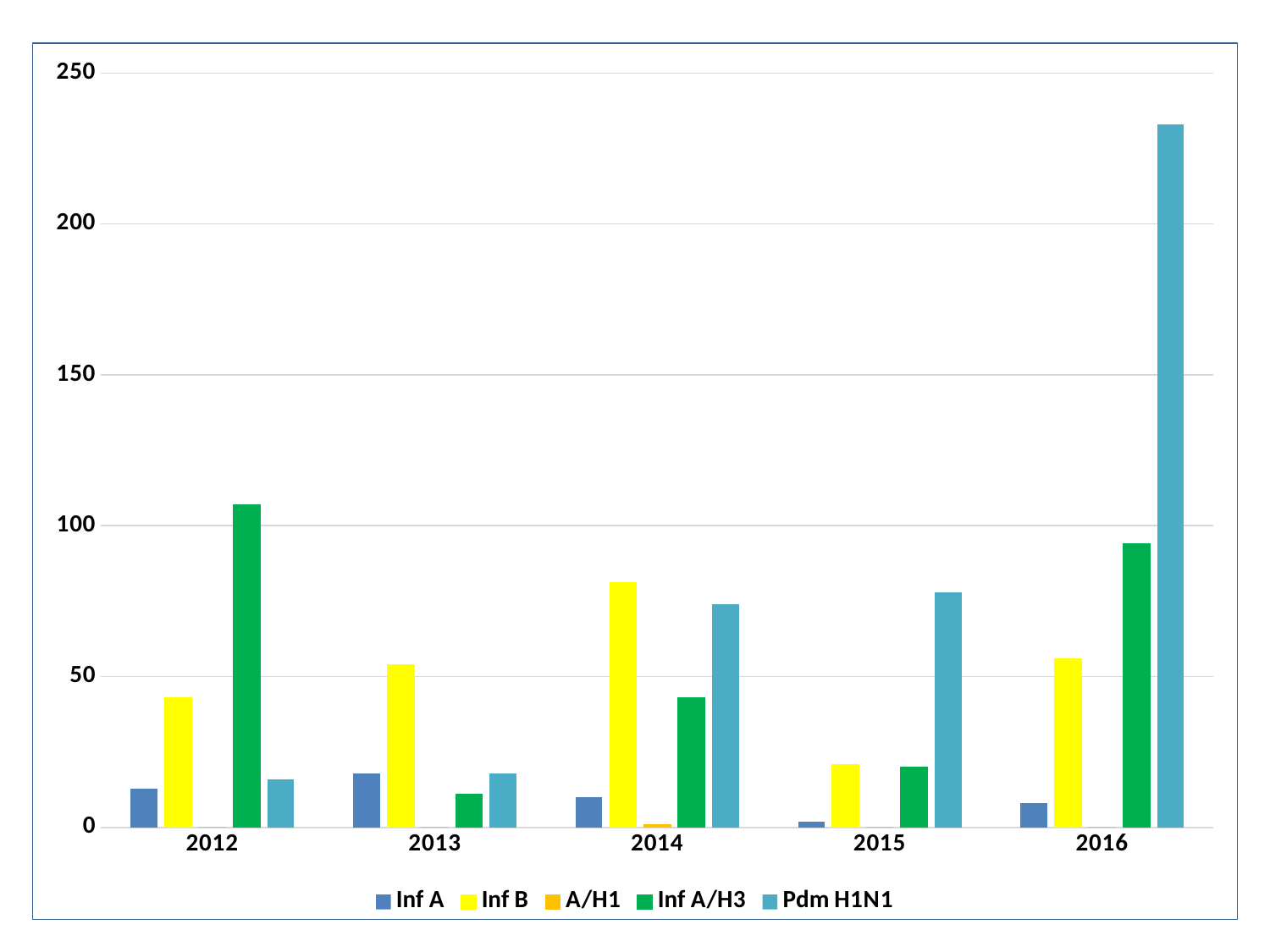

### Chart
| Category | Inf A | Inf B | A/H1 | Inf A/H3 | Pdm H1N1 |
|---|---|---|---|---|---|
| 2012 | 13.0 | 43.0 | 0.0 | 107.0 | 16.0 |
| 2013 | 18.0 | 54.0 | 0.0 | 11.0 | 18.0 |
| 2014 | 10.0 | 81.0 | 1.0 | 43.0 | 74.0 |
| 2015 | 2.0 | 21.0 | 0.0 | 20.0 | 78.0 |
| 2016 | 8.0 | 56.0 | 0.0 | 94.0 | 233.0 |

Supplement: S1 Fig — (PPTX) [file pone.0219376.s003.pptx]
